# The effects of therapeutic horticulture on student well-being and academic resilience

**DOI:** 10.3389/fpsyg.2025.1619827

**Published:** 2025-10-09

**Authors:** Elizabeth R. M. Diehl, David C. Diehl, Siang Yu Tham

**Affiliations:** ^1^Department of Environmental Horticulture, University of Florida, Gainesville, FL, United States; ^2^Department of Family, Youth and Community Sciences, University of Florida, Gainesville, FL, United States

**Keywords:** therapeutic horticulture, college student mental health, academic resilience, student stress, student anxiety, student wellness, student resilience

## Abstract

**Introduction:**

College students across the United States are struggling with significant and increasing levels of stress and anxiety, which contribute to both personal and academic distress. There is a growing need to develop and evaluate programs to reduce stress and anxiety and build resilience in students. The focus of this study was to explore the use of therapeutic horticulture (TH) with university students, specifically hypothesizing that participants would experience reductions in stress and anxiety and increases in resilience and academic resilience after participating in the program.

**Methods:**

Fifty-one university students participated in at least eight weeks of therapeutic horticulture programming and completed pre- and post-surveys assessing participants’ perceived stress, state and trait anxiety, general resilience, and academic resilience. Paired sample t-tests were computed to determine if there was significant change on each outcome variable from the pre-survey to the post-survey.

**Results:**

Significant pre-post improvements in outcomes were found for: academic resilience, including perseverance, help seeking, and managing negative emotions; perceived stress; state and trait anxiety; and general resilience. Findings indicate that therapeutic horticulture is potentially beneficial across a variety of relevant outcomes.

**Discussion:**

The findings indicate that therapeutic horticulture is a promising intervention model for improving mental health and academic outcomes for U.S. college students. These outcomes are critical for overall health and well-being, as well as academic performance, which contributes to lifelong positive outcomes. Future research should include strong study designs that include random assignment into conditions.

## Introduction

College students across the United States are challenged by significant and increasing levels of stress and anxiety. The Healthy Minds Network (2021) reported that more than 60% of college students met the criteria for at least one mental health problem during the 2020–2021 academic year, up almost 50% from 2013 (Lipson et al., 2022). Fifty percent of students either agreed or strongly agreed that they needed assistance with emotional or mental health issues. Just 16% of students were currently receiving therapy or counseling (Healthy Minds Network, 2021). The 2024 State of Higher Education Report stated that one in three students currently enrolled in college considered dropping out and of those, 64% cited emotional stress or mental health concerns (Gallup-Lumina Foundation, 2024).

Psychological distress, in turn, can impact academic success. A recent study reported that 53% of students experiencing anxiety, 54% experiencing stress, and almost 61% experiencing depression stated that it negatively impacted academic performance (ACHA, 2022). In addition to stress and anxiety, 80% of college students may experience loneliness during their college experience (Arnett, 2000). Negative outcomes for young adults experiencing loneliness include susceptibility to illness (Miller, 2011), more alcohol consumption (Arpin et al., 2015), poor sleep quality (Segrin and Burke, 2014), poor college performance (Wohn and LaRose, 2014), and increased risk of suicide (Chang et al., 2015).

These mental health challenges are entry points for a vicious cycle of worsening mental health. Depression and anxiety can lead to social withdrawal which increases the risk of loneliness and social isolation (Luo, 2023). Likewise, loneliness and social isolation are predictors for developing anxiety and depression and can contribute to their increase over time (U.S. Surgeon General, 2023). A systematic review found that children and adolescents experiencing social isolation and loneliness are at an increased risk of anxiety and depression which remains high up to nine years later (Loades et al., 2020). Similarly, researchers found a positive correlation between anxiety symptoms and academic stress in college students and that academic stress is a risk factor for anxiety symptoms (Beiter et al., 2015). Additionally, anxiety affects and is impacted by physical health (WHO, 2023).

### Stress

Stress is often defined as the psychological and/or physiological response to perceived threats or other internal/external stressors (APA, 2018a). It impacts emotions, influences behavior, and encompasses changes that affect almost every bodily system (Anisman and Merali, 1999; APA, 2018a). According to the Spring 2022 National College Health Assessment, 75% of students reported moderate or severe psychological stress. Stress was reported as an academic impediment by 37.3% of all students in 2023 compared to 32% in 2016, representing a 17% increase (ACHA, 2016, 2023). Not only can stress negatively impact academic performance (Córdova et al., 2023), but students experiencing a high level of stress also report reduced enjoyment in learning (Flinn et al., 2016), poorer skill acquisition (Córdova et al., 2023), and decreased memory retrieval (Córdova et al., 2023; Wolf, 2017).

### Anxiety

The American APA (2018b) defines anxiety as an emotion characterized by worry, tension, intrusive thoughts, and physiological changes such as increased blood pressure and heart rate. Related to fear, anxiety tends to be correlated with a future-oriented focus on a broad or diffuse threat. Repeated patterns of anxiety can lead to sleep problems (Dunn et al., 2022), negative health outcomes (Aquin et al., 2017; WHO, 2023), and decreased cognitive performance (Majeed et al., 2023; Hartanto and Yang, 2022). In 2023, 30.4% of students reported having a diagnosed anxiety condition compared to 24.9% in 2016, representing a 22% increase (ACHA, 2016, 2023). Researchers found that high academic distress was associated with a higher probability of student anxiety (Zakeri et al., 2021). Anxiety is also positively associated with student burnout (Abreu Alves et al., 2022), reduced academic engagement (Mou et al., 2022), and lower academic performance (Moreira de Sousa et al., 2018).

### Resilience

Resilience encompasses three key components: an adversity, which constitutes a stressful or traumatic experience; observable signs of healthy functioning following the adversity; and mechanisms that prevent or facilitate recovery from the typical distress. These mechanisms, also referred to as the protective factors, enable the individual to recover from adversity (Hamby et al., 2017). The concept of resilience was originally conceptualized as individual, intrinsic traits such as coping skills and positivity. Further research on resilience increasingly acknowledges the protective role of extrinsic factors as well, including social relationships and resources within one’s environment (Luthar et al., 2000; Van Breda, 2001). Research based in social-ecological models has emphasized that resilience is characterized by complex interactions between the individual and specific characteristics of their environment. Researchers have proposed models that explore the qualities of different contexts that help facilitate resilience (Masten et al., 2023; Ungar, 2011; Ungar et al., 2013).

College is a time of significant transition to young adulthood (Walters et al., 2018), which can be challenging and stressful for many students as they experience changes in their environment, social and family relationships, education, responsibilities, and finances, among other areas. Resilience has been demonstrated to be an effective mechanism in empowering students to navigate these challenges, particularly in reducing stress and alleviating depressive symptoms (Smith and Yang, 2017). Despite these benefits, a meta-analysis by Chua et al. (2022) found that globally, 36% of college students reported low resilience. Students exhibiting low levels of resilience demonstrate poorer well-being, increased stress levels, a higher likelihood of dropping out of school (Ang et al., 2022; Sosu and Pheunpha, 2019), decreased academic motivation (Pascoe et al., 2019), impaired memory retrieval (Trammell and Clore, 2014), and poor academic performance (Chua et al., 2022). A study of first- and second-year medical students revealed that general distress and lack of adaptive coping strategies were associated with burnout (Abreu Alves et al., 2022). The study also reported that academic engagement served as a protective factor, reducing the effect of burnout on dropout intention.

### Academic resilience

Because education is a primary focus of the college experience, an area of resilience that is of particular interest is that of academic resilience. Academic resilience refers to the capacity to return to healthy academic or educational functioning following an adversity (Cassidy, 2016). Several risk factors may influence an individual’s academic performance including stress, anxiety, depression, and loneliness (Wohn and LaRose, 2014), as well as chronic illness, undesirable living conditions or homelessness, experiencing a natural disaster, and facing financial hardships among others (Horton, 2015; Masten, 1994; Sosu and Pheunpha, 2019; Yang and Wang, 2022). While numerous studies have been conducted on resilience and protective factors in mental health, research in protective factors influencing academic resilience remains limited.

Although the reasons for the dramatic increase in mental health challenges among college students during the past decade are not fully understood, contributing factors may include perceived increases in parental expectations (Curran and Hill, 2022), impacts of social media and social ‘worth’ (Powdthavee, 2014; Scelfo, 2015), financial stressors (McAlpine, 2021; NASPA, 2023), shifting societal values (McAlpine, 2021), and more recently, the trauma of Covid-19 (Kim et al., 2022; Lipson et al., 2022). On the more positive side, the awareness of mental health issues and reduction in the associated stigma may allow more students to voice their struggles (McAlpine, 2021; NASPA, 2023). Additionally, advances in treatment and medications have enabled more students with mental illness to go to college (Abrams, 2020; Biancolli, 2021).

College counseling and wellness centers everywhere are overwhelmed with almost 90% of center directors reporting an increase in the number of students seeking services in 2019 (LeViness et al., 2019). This high demand leads to longer wait times for students, mounting pressure on counseling centers, and counselor burnout (Abrams, 2020; Biancolli, 2021). However, colleges have unique access to students and therefore have greater opportunity to provide resources and additional interventions to those in need. As Lipson et al. (2022) point out, colleges and universities are ideal settings to approach mental health issues during a significant stage of life.

## Therapeutic horticulture

Therapeutic Horticulture (TH) is a process that uses structured horticultural activities as a therapeutic modality to support program and health goals and improve well-being through active and passive participation (AHTA, 2013). This process was described as early as the 19th century by Dr. Benjamin Rush, recognized as the ‘father’ of American psychiatry. He was the first to document the positive effect that working in a garden had on individuals with mental illness (Monroe, 2015). Horticultural activities have long-proven therapeutic benefits for people experiencing mental health issues and have been associated with significant improvements in quality of life, well-being, social relations, and physical and cognitive outcomes (Elsadek et al., 2019; Gonzalez et al., 2010; Shao et al., 2020). Additional studies have found horticulture interventions led to significant decreases in stress (Egerer et al., 2022; Kaplan, 1995; Ulrich et al., 1991), anxiety (Gonzalez et al., 2010; Son et al., 2004; Yang et al., 2022), and social isolation (Howarth et al., 2020). Therapeutic horticulture has also been shown to boost mood (Elsadek et al., 2019), life satisfaction (Son et al., 2004), and self-esteem (Sonti and Svendsen, 2018; Wood et al., 2016), enhance interpersonal relationships (Waliczek et al., 2005) and improve body image (Swami et al., 2020). Because TH uses plants and nature as a vehicle for exploring mental health issues, there may be less stigma and/or cultural barriers associated with participation.

While substantial research has been conducted on the mental health and stress reduction benefits of therapeutic horticulture interventions on other populations including children, adults, and seniors (Lu et al., 2023; Rosa et al., 2023), few studies have been published about these benefits for college students. However, some benefits from exposure to gardens and greenspace have been reported for college students, including lower perceived stress (Holt et al., 2019; Larson et al., 2022; Macchi and Coccia, 2022; Matias et al., 2023; Mecham and Joiner, 2021), increased positive emotions and well-being (Baur, 2020; Holt et al., 2019; Mecham and Joiner, 2021; Stepansky et al., 2022), improved self-efficacy (Macchi and Coccia, 2022), as well as improved academic self-efficacy (Guo et al., 2023). These benefits were generally greater for those actively participating in the garden or in a horticultural activity.

## Aim of study

This study sought to understand the role that therapeutic horticulture (TH) could play in improving mental health and academic outcomes in students at the University of Florida. The specific hypotheses were that participants would experience reductions in stress and anxiety and increases in resilience and academic resilience after participating in the program.

## Materials and methods

### Selection and recruitment of participants

Recruitment for this study took place via social media posts, campus flyers, email announcements, and information provided at the UF Counseling & Wellness Center (CWC). Interested students were invited to email or call the study PI. During that initial contact the PI and/or study staff inquired about student status. If the individual was a current student at UF they were invited to join the group and informed of the research study. Students were not required to take part in the study to join the TH group but were invited to join the study if there were at least eight weeks remaining in the current semester.

Eighty-five participants from the student body of the University of Florida consented to participate in this research study (IRB# 202101905). The study took place in three phases over three semesters; Fall 2021, Spring 2022, and Fall 2022. Fifty-one participants who attended at least eight sessions and completed both the pre-survey and post-survey are included in the analyses here.

### Therapeutic horticulture program protocol

Students were admitted to the TH group on a rolling basis if there was space but invited to participate in the study only if there were at least eight sessions remaining in the semester. In addition, some students declined to participate in the research study so there was a mix of research and non-research participants in each group. All participants took part in the same activities no matter their status.

Sessions were offered at different times during the week (mornings and afternoons) to accommodate academic schedules. Fall cohorts began in September and ended in December. Spring cohorts began in January and ended in April. Each session lasted 75 min and followed the same schedule, which included 15 min of ongoing propagation work in a mini tabletop greenhouse, 20 min exploring a wellness strategy, and 40 min engaged in a horticulture activity. The structure and schedule of each session was the same throughout the groups. By the end of their eighth session, each participant had been introduced to eight different wellness strategies and participated in eight different horticulture activities. The underlying goals of each session were to: (1) build skills and confidence in working with plants; (2) introduce and practice a wellness strategy; (3) increase understanding of the role that plants and nature can play in personal wellness; and (4) build community among participants.

Each group met in the same greenhouse located on the University of Florida campus in Wilmot Botanical Gardens. The greenhouse is a 2,700 sf conservatory-style glass greenhouse with bathrooms, accessible features, and a fully air-conditioned reception area connected to the greenhouse. The greenhouse has a large area with tables and chairs where a group can work and interact comfortably. Most of the horticulture activities took place in the greenhouse while most of the wellness strategies were explored in the quieter reception area.

The greenhouse sits in the SW corner of the 4.8-acre gardens which contain a variety of garden features and plant types in both sunny and shady areas. The garden was used as an integral part of several horticulture activities such as collecting natural materials to use in projects. A few wellness strategies also took place in the gardens such as mindful walking.

### Session content

Participants were assigned to a TH group with 6–12 other participants based on the group that worked best for their schedule. TH sessions were facilitated by a professionally registered horticultural therapist and a program manager with training in the use of horticulture as a therapeutic modality.

During each session, participants explored a wellness strategy, led by a counselor from the Counseling & Wellness Center at UF, and then engaged in a related horticulture activity. Wellness strategies included mindfulness, cognitive defusion, anxiety reduction techniques, and self-compassion strategies, among others. Horticulture activities included hands-on basic plant care such as plant propagation techniques and methods, proper planting and maintenance, and general plant identification and knowledge. Participants also participated in plant art activities and regularly took plants and plant crafts home.

### Study design and data collection

This study used a single-group, non-experimental pre−/post-survey method. At the first visit, participants completed the informed consent. After signing the ICF, they filled out a demographic questionnaire inquiring about their age, race/ethnicity, gender, academic major, and gardening experience, among other information. Participants then filled out a set of four self-report psychometric questionnaires and then immediately joined the TH session with the rest of the group.

The therapeutic horticulture sessions ran for 12–13 weeks each semester. Study participants were asked to attend sessions once per week. If a participant was not able to attend at least eight sessions, their participation in the study was discontinued. The participant could continue attending the sessions, but any data collected was not used in final data analyses. At the participant’s eighth visit, following the scheduled TH activity, they completed the same four psychometric surveys as during their first visit. Participants who completed their eighth visit and corresponding questionnaires could continue to attend the remaining sessions, but no further data was collected from these individuals.

### Measures

*Stress.* Participants’ perceived stress was measured using the Perceived Stress Scale (PSS) (Cohen et al., 1983), which is based on a 5-point Likert Scale and is the most widely used psychometric tool to gauge the perception of stress. This study used the validated 10-item Perceived Stress Scale, which has strong validity and reliability (Roberti et al., 2006).

#### Anxiety

State and trait anxiety were assessed using the State–Trait Anxiety Inventory Short Form (STAI-6) (Spielberger et al., 1983) which seeks to gauge anxiety levels. This leading measure of personal anxiety differentiates between the temporary condition of *state* anxiety and the more general or persisting *trait* anxiety using a 4-point Likert scale. The short-form version of the STAI has been validated as a reliable alternative to the full scale (Zsido et al., 2020).

#### General resilience

General resilience was measured using the Brief Resilience Scale (BRS) (Smith et al., 2008). This six-item scale includes both positively and negatively worded statements that are responded to using a 5-point Likert scale. The scale assesses one’s ability to bounce back or recover from stress.

#### Academic resilience

The Academic Resilience Scale (ARS-30) was used to explore process rather than outcome aspects of resilience (Cassidy, 2016). This 30-item, vignette-based scale measures academic resilience based on specific adaptive behavioral and cognitive-affective responses when confronted with academic adversity.

### Data analysis

All outcome measures were treated as continuous variables and paired sample t-tests were computed to determine if there was significant change on each outcome variable from the pre-survey to the post-survey. All analyses were conducted using the Statistical Package for the Social Sciences (SPSS), version 29.

### Participant profile

Fifty-one individuals, who completed eight therapeutic sessions and also completed the pre-survey and post-survey, are included in this study. Study participants were currently registered students at the University of Florida, representing 34 different majors. The participants ranged in age from 18–53, with a median age of 22. This includes roughly equal numbers of undergraduate (52.9%) and graduate students (47.1%). About 71% of participants identified as female, 24% identified as male, and 6% identified as queer / gender nonconforming. Demographic and background characteristics are reported in Table 1.

**Table 1:** Participant characteristics.

Variable	Value	*N*	%
Full sample		51	100
Age (median = 22)	18–21	21	41.2
	22–25	14	27.5
	26–30	7	13.7
	31–53	9	17.6
Level of study	Undergraduate	27	52.9
	Graduate	24	47.1
Gender	Male	12	23.5
	Female	36	70.6
	Gender queer/nonconforming	3	5.9
Race	White	30	58.8
	Black/African American	2	3.9
	Asian	7	13.7
	No Answer	12	23.5
Ethnicity	Hispanic	13	25.5
	Not Hispanic	38	74.5
Housing	On campus	8	15.7
	Off campus	43	84.3
Experience with outdoor gardening	Never	18	35.3
	In the past	23	45.1
	Currently	10	19.6
Experience with indoor plant care	Never	12	23.5
	In the past	11	21.6
	Currently	28	54.9
Level of gardening/plant care experience	None	8	15.7
	Some	37	72.5
	Extensive	6	11.8

## Results

The analysis of change from pre-survey to post-survey revealed significant positive change for all outcomes measured. For academic resilience, the total score improved from pre-test to post-test, t(50) = 6.81, *p* < 0.001, as did the three subscales: perseverance, t(50) = 5.92, *p* < 0.001; help seeking, t(50) = 4.88, *p* < 0.001; and negative emotions, t(50) = 5.05, *p* < 0.001. Effect sizes for these improvements ranged from 0.61 to 0.87. On the measure of perceived stress, mean scores were significantly reduced from pre to post, t(50) = 4.62, *p* < 0.001, with an effect size of 0.62. Similarly, measures of state anxiety, t(50) = 8.34, *p* < 0.001, and trait anxiety, t(50) = 3.75, *p* < 0.001, were also significantly reduced, with effect sizes of 1.18 and 0.53, respectively. General resilience significantly increased, t(50) = 2.61, *p* = 0.011, with an effect size of 0.33. See Table 2 for detailed descriptive and inferential statistics.

**Table 2:** Description of findings for change over time on all outcome variables.

Outcome variable	Reliability (*α*)	Pre-test M (SD)	Post-test M (SD)	(*t*, 50df)	*p*	Effect size (*g*)
Academic resilience scale – Total	0.886	100.7	111.6	6.325	<0.001	0.87
ARS – Perseverance	0.794	51.8	56.4	5.933	<0.001	0.82
ARS – Help seeking	0.711	32.4	35.0	4.398	<0.001	0.61
ARS – Negative emotions	0.802	16.5	20.2	5.467	<0.001	0.75
Perceived stress scale	0.787	22.4	17.7	4.500	<0.001	0.62
General resilience	0.898	17.8	19.0	2.362	0.011	0.33
State–Trait anxiety inventory – State anxiety	0.714	13.7	9.9	8.587	<0.001	1.18
State–Trait anxiety inventory – Trait anxiety	0.728	15.8	14.2	3.835	<0.001	0.53

^1^Reported *p* values are 1-sided because all hypotheses were directional, hypothesizing positive program effects.

## Discussion

Confirming the study’s primary hypothesis, participation in the therapeutic horticulture program was associated with a significant increase in students’ academic resilience, a finding with a large effect size of 0.87. Furthermore, improvements in the sub-scores of perseverance, reflecting and adaptive help-seeking, and avoidance of negative affective and emotional response were reported, suggesting a positive impact on academic life. Positive outcomes related to academic resilience are especially important in college student populations because of the centrality of educational success in student life. Further, academic resilience has been linked to positive outcomes such as enjoyment of school, participation in class, and general self-esteem (Martin and Marsh, 2006). Elnaem et al. (2024) also found that academic resilience correlates with positive academic outcomes, with coping strategies and social support playing critical roles in influencing how students manage academic adversity.

Scores on the Brief Resilience Scale, which measures general resilience, also indicate a statistically significant improvement, with an effect size of 0.33. Considerable research shows that resilience has protective effects on mental well-being and plays a mediating role in state and trait anxiety (Bacchi and Licinio, 2017; Peng et al., 2022). Resilient students have the adaptive ability to overcome adversity and anxiety in times of heightened challenge (Devi et al., 2021).

A statistically significant reduction of both state and trait anxiety was found as demonstrated by the scores on the STAI-6. These findings suggest that therapeutic horticulture is effective in alleviating both temporary and persisting conditions of anxiety. These results parallel previous studies which demonstrate the effectiveness of therapeutic horticulture in significantly reducing anxiety among other groups (Gonzalez et al., 2010; Son et al., 2004; Yang et al., 2022).

The scores on the Perceived Stress Scale revealed significant reduction in perceived stress, which suggests a positive effect of TH interventions on reducing stress. These results support studies with other groups on the effectiveness of a TH intervention on stress (Egerer et al., 2022; Kaplan, 1995; Ulrich et al., 1991). In an academic setting, these findings are significant since stress is a risk factor for poor academic performance (Wohn and LaRose, 2014), and more specifically, decreased memory retrieval (Wolf, 2017), poorer skill acquisition (Córdova et al., 2023), and reduced enjoyment in learning (Flinn et al., 2016). Reducing stress and the negative emotions that often accompany stress may lead to more effective learning. Perhaps the intentional community-building efforts in this study led to increased social support among participants. Kugbey et al. (2015) reported a significant negative correlation between social support and stress in academia; when social support increases, stress decreases. The feeling of being supported is crucial in helping students manage their stress levels and shield them from various psychological issues (Camara et al., 2017).

### Limitations/improvements

This study has several limitations. First, there was no random assignment in this study. Participants signed up for the study knowing there were horticulture activities involved. Therefore the sample may be subject to selection bias since it is possible that some participants may have had a positive attitude toward the intervention. Second, the study did not include a control group to determine if the reported changes were in fact due to the TH intervention or other external influences during the semester. Third, all outcome measures used (ARS, PSS, BRS & STAI-6) rely on self-reported data, which could be influenced by social desirability bias, leading participants to over-report improvements or under-report negative emotions. Fourth, while race was not a focal variable of this analysis, the relatively high frequency of ‘No answer’ responses on the race question makes it more challenging to fully describe the characteristics of this sample. Lastly, the participants were all students enrolled at a single large public university and therefore the findings may not be generalizable to students at other institutions. Nevertheless, this research offers valuable insight into the potential benefits of therapeutic horticulture and serves as an important foundation for future studies on the impacts of TH on the mental health and academic resilience of college students.

## Conclusion

In this study, a structured therapeutic horticulture program led to significant improvements in general and academic resilience and reductions in stress and anxiety. These findings are noteworthy given the scarcity of research in this area and they contribute to a better understanding of the connections between plants, nature, and student mental health. This study points to an expanded, more holistic paradigm of addressing student mental health, one that moves beyond a sole focus on the university counseling center. There are many students whose mental health needs may be met by accessible, alternative routes to professional intervention and these findings support that possibility. Therapeutic horticulture programs may reduce the burden on university counseling centers by engaging students who are not yet in acute need of services, but are experiencing increasing academic and mental health distress. By sharing experiences and working together on intrinsically meaningful horticulture-based activities, students in this program developed community and social support while learning coping strategies. Through this process students can improve confidence, self-efficacy, and self-worth, strengthening their overall health and wellness and boosting academic resilience.

## Data Availability

The datasets in this article are not readily available because that is not a condition in our IRB contract. Questions about the dataset should be directed to leahdiehl@ufl.edu.
